# The urgency of ensuring equitable and improved access to oral health care during the Coronavirus Disease 2019 pandemic: The case of Peru

**DOI:** 10.34172/jrhs.2021.60

**Published:** 2021-08-28

**Authors:** Akram Hernández-Vásquez, Diego Azañedo

**Affiliations:** ^1^Centro de Excelencia en Investigaciones Económicas y Sociales en Salud, Vicerrectorado de Investigación, Universidad San Ignacio de Loyola, Lima, Peru; ^2^Universidad Científica del Sur, Lima, Peru

## Dear Editor-in-Chief

The 74^th^ World Health Assembly has recently approved the resolution called “Achieving better oral health as part of the universal health coverage and non-communicable disease agendas towards 2030”^1^. In this framework, the General Director of the World Health Organization (WHO) was asked to develop a global strategy to address oral diseases as part of an action plan to be executed in 2023 with the best interventions in the fight against these diseases that affect more than 3.5 billion people^2^.

The resolution on oral health recommends a change in dental care from the traditional curative approach to one that is preventive and emphasizes the need for timely, comprehensive, and inclusive dental care within the primary health care system, in light of the great inequalities present in the marginalized and low-income populations, especially in low- and middle-income countries^2^. In this sense, the member states of the WHO have a difficult mission to achieve in terms of guaranteeing access to dental services, which have been seriously affected by the COVID-19 pandemic due to the collapse of health services, the restriction of oral care to urgencies and emergencies, and fear of contagion by the population^3^. This complex scenario may have affected low- and middle-income countries to a greater extent, increasing inequalities in access to oral health services within them. This is especially important in Peru, which is one of the countries hardest hit by the pandemic^4^.

**Figure 1:**
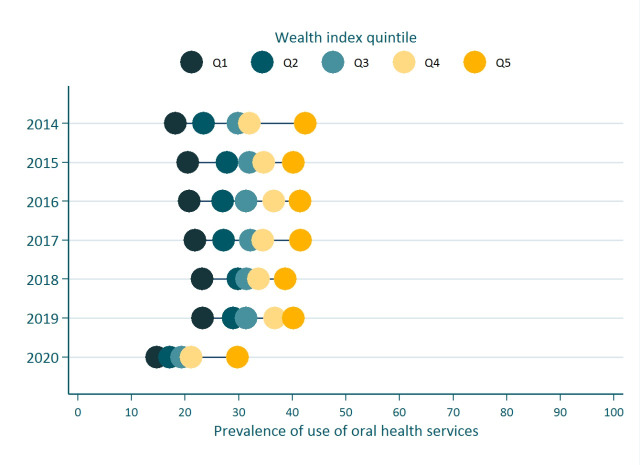
Trends in the prevalence of use of oral health services according to the quintile of wealth index in children under 12 years of age in Peru.

Here, we report the prevalence of use of dental services in the last six months according to the quintile of wealth index (Q5 representing the wealthiest and Q1 the poorest)^5^ in children under 12 years of age in Peru based on the Demographic and Family Health Surveys from 2014 to 2020. The prevalence of use of dental services in 2014 in this population was 27.6%, while in 2020, it was 19.6%. Figure 1 shows that the use of dental services in the population of quintile 1 had grown by approximately 5 percentage points during 2014-19 (from about 20% to almost 25%); however, this progress has notably decreased in 2020, during which only 15% of children under 12 years of age attended a dental service in the last six months. This reduction is also notable in quintiles 3 and 4 with 12 and 16 percentage points of difference between the years 2019 (Q1: 23.3%; Q2: 29.0%; Q3: 31.4%; Q4: 36.7%; Q5: 40.2%) and 2020 (Q1: 14.7%; Q2: 17.1%; Q3: 19.3%; Q4: 21.1%; Q5: 29.8%).

 The neglect of oral diseases and even more of the promotion and prevention of oral health through the use of dental services can have adverse consequences on population health and the economy of the countries. In this sense, we believe that the incorporation of oral health into the WHO agenda is timely; however, the figures on the use of dental services before and after the pandemic demonstrate that we cannot wait until 2023 to take action. For this reason, the WHO and the member states must prioritize the urgent establishment of strategic plans for the prevention and promotion of oral health with emphasis on increasing the use of this essential health service and to mitigate the impact of the pandemic in the population oral health. Some of the measures that could be taken into account include 1) Enhancement of infection and control measures in oral health settings in order to guarantee a safe dental attendance, 2) Implementation of massive use of the media to deliver clear and concise information on preventive oral health methods and education in oral health, 3) Strengthening the use of tele-dentistry in remote and hard-to-reach areas, and 4) Delivery of health promotion messages that address common risk factors for oral and general chronic diseases in order to avoid duplication of efforts.

## Acknowledgments

 The authors are grateful to Donna Pringle for reviewing the language and style.

## Conflict of Interest Statement

 The authors declare no competing conflict interests.

## Funding

 Self-funded.
